# Towards personalized chemotherapy of acute lymphoblastic leukemia

**DOI:** 10.18632/oncotarget.26420

**Published:** 2018-12-04

**Authors:** Aleš Hnízda, Tai Yang

**Affiliations:** Aleš Hnízda: Department of Biochemistry, University of Cambridge, Cambridge, UK

**Keywords:** relapsed leukemia, chemotherapy resistance, thiopurine, purine metabolism, personalized therapy

Thiopurines, including mercaptopurine and 6-thioguanine, are chemotherapeutics commonly used for treatment of acute lymphoblastic leukemia (ALL). Due to genetic instability of leukemia cells, efficiency of thiopurine treatment decreases over time that can lead to relapse of the disease. Among others, relapse-specific mutations in PRPS1 and NT5C2 were identified as important drivers for chemoresistant ALL [1, 2]. These genes are responsible for maintaining homeostasis of purine metabolism and are essential for conversion of thiopurines to pharmacologically active species. Recent studies showed that mutations in both genes act through a shared molecular mechanism, which opens new opportunities for targeted antileukemic therapy.

Biochemical examinations revealed that missense mutations in PRPS1 and NT5C2 cause misregulation of respective enzymes leading to their constitutive hyperactivity [1, 3]. PRPS1 mutants have impaired responsivity for allosteric inhibition *via* end-products of the purine biosynthetic pathway (GDP or ADP), while NT5C2 variants are constitutively active without a need for activating compounds such as ATP. Structurally, mutations in both PRPS1 and NT5C2 affect oligomerization interfaces, demonstrating their importance in regulatory movements of the proteins. Since the mutations in NT5C2 are heterozygous, enzymes are assembled into heteromeric complexes containing both wild-type and mutant subunits [4]. Notably, the intersubunit motions stimulate even the wild-type polypeptide showing that the heteromeric proteins have distinct properties from their individual components. These finding highlights protein complexes of variable composition as relevant drug targets for individualized therapy. However, structural studies of such mixtures are extremely challenging as traditional techniques (X-ray crystallography and NMR spectroscopy) usually require homogenous samples. Current obstacles might be overcome by a rapid progress in single-particle cryo-EM that enables 3-D classification to identify different protein conformations. This approach represents an important direction in structure-based drug discovery with respect to patient genotype.

**Figure 1:**
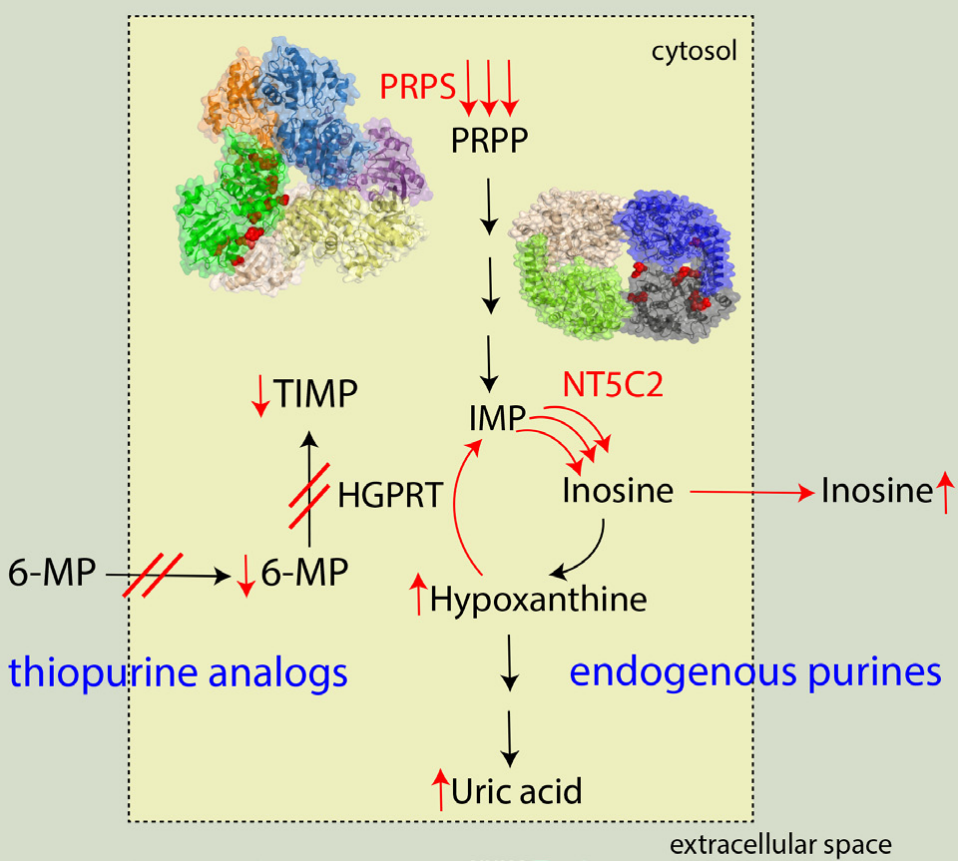
Molecular mechanism of chemoresistant acute lymphoblastic leukemia due to activating mutations in PRPS1 and NT5C2 Mutations (highlighted as red spheres) are distributed at oligomeric interfaces; each subunit in the protein complexes is depicted in different colour. Hyperactivation of both enzymes causes purine overproduction that suppresses transport and intracellular metabolism of thiopurine analogs.

To explore therapeutic approaches alternative to direct inhibition of the hyperactive variants, previous works have focused on the metabolic changes caused by PRPS1 and NT5C2 mutations [1, 5]. Hyperactive variants in both PRPS1 or NT5C2 lead to overproduction of purines and their increased secretion to extracellular space which blocks cellular transport of thiopurines and their metabolic activation *via* hypoxanthin-guanin phophoribosyltransferase (HGPRT; Figure 1). Misregulation of the entire purine metabolism confers chemoresistance of leukemia cells toward thiopurines; however, it also decreases their fitness under normal conditions. This makes the cells more sensitive to pharmacological modulation of the purine metabolic pathway, as has been demonstrated by selective cytotoxicity of IMPDH inhibitors in leukemic cells carrying NT5C2 mutations [5]. In principle, additional enzymes in purine metabolism could be suitable targets in chemoresistant ALL. For instance, inhibitors against tri-functional GART (a core enzyme of de novo purine synthesis) or purine nucleoside phosphorylase (an enzyme upstream of NT5C2) have been clinically tested for various diseases that can be evaluated in a relapsed ALL context.

An attractive direction for future studies might be the targeting of purinosome, a metabolon composed of enzymes from the purine de novo synthesis. Interestingly, purinosome formation is affected in various disorders of purine metabolism, including inherited defects of purine de novo synthesis and Lesch-Nyhan syndrome (HGPRT deficiency) [6, 7]. Purinosome assembly and its subcellular localization are regulated *via* multiple mechanisms such as cell cycle-dependent activity of casein kinase 2 and mTOR signalling [8]. It is tempting to speculate that modulation of purinosome assembly might provide novel opportunity for targeted anticancer therapies.

In summary, studies on PRPS1 and NT5C2 in chemoresistant leukemia elucidated a complex regulatory network in the purine metabolic pathway. In addition, multiple novel mechanisms have been recently described in distinct pathological states. For instance, PRPS1 is also stimulated by excessive phosphorylation at oligomerization areas in hepatocarcinoma [9]. Taken together, emerging mechanisms might be exploited for development of individualized treatment depending on specific diagnosis and genotype of patients.
